# The TNF-**α** -308 Promoter Gene Polymorphism and Chronic HBV Infection

**DOI:** 10.1155/2012/493219

**Published:** 2012-10-24

**Authors:** Sirous Tayebi, Ashraf Mohamadkhani

**Affiliations:** Digestive Disease Research Centre, Tehran University of Medical Sciences, Shariati Hospital, North Kargar Ave, Tehran 14114, Iran

## Abstract

*Background and Aims*. TNF-**α** -308 allele promoter polymorphism has been known to be a potential prognostic factor in patients with chronic HBV infection. We tried to determine how TNF-**α** -308 allele promoter polymorphism would affect the prognosis in patients with chronic HBV infection. *Methods*. We searched MEDLINE, EMBASE, and reference lists of relevant review articles related to the association between “TNF-**α** G-308A promoter polymorphism” with “chronic HBV infection”. We only focused on searching -308 locus in published studies. We reviewed 21 original articles about TNF-**α** -308 allele polymorphism and its effect on prognosis in patients with chronic HBV infection and discussed the results. *Results*. conflicting results were observed. The results were divided into 3 groups including neutral, negative, and positive associations between TNF-**α** -308 allele polymorphism and prognosis in patients with chronic HBV infection. We summarized the primary data as a table. *Conclusions*. Authors concluded that although there is an upward trend in evidence to claim that there is a positive relation between TNF-**α** G-308A promoter polymorphisms and resolution of chronic HBV infection, due to many biases and limitations observed in reviewed studies, an organized well-designed study is needed for clarifying the real association.

## 1. Introduction

It is believed that during chronic hepatitis B infection, the host immune response is responsible for both hepatocellular damage and viral clearance [1, 2]. Hepatocyte damage persuades an inflammatory response through activation of tissue macrophage Kupffer cells [3]. These activated cells secrete antiviral cytokines which is thought to be central in suppression or clearance of HBV from the infected liver [4]. Cytokines are proteins or glycoproteins produced by cells acting on their specific receptors on the other cells' surfaces. They are central mediators of inflammatory events such as infection or peripheral trauma. Several cytokines have been identified that participate in the process of viral clearance via host immune response to HBV. They include TNF-*α*, TGF*β*, PGF, and other factors contributing towards the fibrogenesis [5, 6]. Among these, TNF-*α* is the most important cytokine in host immune response to viral infection [7, 8]. TNF-*α* is a pleiotropic cytokine, located 850 kb telomeric of class II HLA-DR locus of the short arm of chromosome 6, which induces cellular responses such as proliferation, production of inflammatory mediators, and cell death [9]. In the liver, TNF-*α* is involved in pathophysiology of viral hepatitis, alcoholic liver disease, nonalcoholic fatty liver disease and ischemia-reperfusion (I/R) injury. This cytokine shows a remarkable functional duality; it is not only a mediator of hepatotoxicity but also an inducer for hepatocyte proliferation and liver regeneration. TNF-*α* is produced mainly by macrophages and also by a broad variety of other cell types including lymphoid cells, mast cells, endothelial cells, fibroblasts, and neurons [9]. Circulating TNF-*α* level increases during HBV infection [10]. Increased hepatic level of TNF-*α* is associated with suppression of HBV replication in transgenic mice which expresses HBV in the liver [9]. TNF-*α* inhibits HBV replication by noncytopathic suppression mediated by NF-*κ*B pathway [11].

The way that TNF-*α* inhibits HBV replication differs from other cytokine inhibitors because it targets the stability of nascent nucleocapsids. The maintenance of the cccDNA pool is thought to be critical for HBV persistence in infected hepatocytes and TNF-*α* mediated decline of nuclear cccDNA levels may be via preventing the formation of nucleocapsids that delivers cccDNA to the nucleus [7]. Type I IFNs likely suppress HBV mRNA transcription and type II IFNs might regulate the activity of La proteins, which may play a putative role in HBV mRNA stability [35]. TNF-*α* might also require both proteasome activity and iNOS activity [36]. TNF-*α* has also been shown to be effective in angiogenesis processes. Neoangiogenesis in the liver of HBV-infected patients suggests that TNF-*α* might also have a role in the development of viral hepatitis-associated liver tumors [37]. Locus -308 has been much more considered than any other loci (-238, -863) in correlation between genetic materials and clinical manifestation. The results from other loci in correlation with chronic HBV infection or other diseases from different studies were not highly significant. Although one study has claimed that TNF-*α* 238A allele may increase the risk of chronic HBV infection in European populations [38], when seeking for correlation of this locus with cancer, the results are not significant [39]. When comparing these results with studies about locus -308, we found that not only is locus -308 important in breast cancer but also in association with other diseases like essential hypertension [40]. Few studies found both -238 and -308 loci significantly important in correlation with diseases [41–43]. Many studies found both -308 and -238 loci nonsignificant when seeking for correlation between clinical manifestations and genetic materials [15, 44–47]. When comparing locus -308 with other loci (-238, -836), the correlation of locus -308 with other diseases such as Guillain-Barré syndrome [48], tuberculosis [49], and ANCA-associated vasculitis [50] has recently been approved. Based on the reasons mentioned above, we were trying to investigate only about correlation of locus -308 with HBV infection (and not with other diseases).

The significant role of tumor necrosis factor (TNF-*α*) in inflammation process has been attracted a great attention in both the regulation of the TNF-*α* gene and the possibility of TNF-*α* variants production. Polymorphisms in particular, at positions -308, are reportedly capable of altering TNF-*α* expression. However, population-based studies have yielded contradictory results regarding the relationships of these polymorphisms with the progression of HBV infection. 

We suppose that binding of cellular factors to TNF-*α* promoter might be influenced by this polymorphism and affects gene expression and disease outcome. For better understanding of association between commonly studied TNF-*α* G-308A promoter polymorphism and susceptibility to chronic HBV infection, we classified the previous findings from all eligible studies.

## 2. Search Strategy

We searched MEDLINE, EMBASE, and reference lists of relevant review articles. All titles and abstracts and original articles of the included studies were independently reviewed by two review authors to see whether the study is discussing about TNF-*α* -308 locus or not. All searches were updated in August 2012.

## 3. Selection Criteria

Any studies about the association between “TNF-*α* gene promoter polymorphism” with “chronic HBV infection” were included. We only focused on -308 locus. This paper does not discuss the other factors affecting prognosis of chronic HBV infection (other loci such -238, -863). This is only a review article to clarify the relation between TNF-*α* -308 gene promoter polymorphism and prognosis in patients with chronic HBV infection, so the other results from studies, not related to -308 locus, were not mentioned in this paper.

## 4. Results

It has always been a challenging field talking about the effects of TNF-*α* -308 gene promoter polymorphisms in chronic HBV infection making us confront with conflicting results. There are 3 different kinds of results about the relationship between TNF-*α* -308 gene promoter polymorphism and prognosis in patients with chronic HBV infection:

The first group of studies is about to say “there is no association”. One study from Japan showed that allelic distributions of both gene promoters (including TNF-*α* and IL-10) were not significantly different between HBV carriers and health volunteers [33]. Another study from Italy demonstrated that TNF-*α* gene promoter polymorphisms do not appear to be determinant of HBV seroclearance [26]. Despite the fact of high prevalence of TNF-*α* gene promoter polymorphism in -308 locus in Iran, it has no association with development of chronic HBV infection [21].

The second groups of studies demonstrates that TNF-*α* -308 gene promoter polymorphisms are associated with either “unfavorable prognosis of chronic HBV infection” or high risk of persistent HBV infection. Korean patients with TNF-*α* -308 gene promoter polymorphisms had higher risks of persistent HBV infection [23]. The genotype -308 G/G and haplotype TCGG are associated with an unfavorable prognosis in patients with chronic HBV infection [26]. In Chinese people, frequency of haplotype GGCCT (-238, -308, -857, -863, -1031) in patients with chronic HBV infection was significantly lower than that in spontaneously recovered group [51]. 

The third group of studies are about to say “there is a positive association between TNF-*α*-308 gene promoter polymorphisms and resolution of HBV infection”. It has been shown that TNF-*α*-308 G/A or A/A promoter polymorphisms are associated with HBV clearance [32]. A meta-analysis about TNF-*α* -308 gene promoter polymorphisms has claimed that TNF-*α* -308 A allele may have a protective effect on the prognosis of chronic HBV infection in Mongoloid populations [52].

As a comprehensive view so far, we summarized all the basic data extracted as a table showing only the number of patients who are different at polymorphism. The most important point about these studies is that they have been designed differently and the methods of the studies vary from “response to special treatment” to “simple study of 2 groups of HBV patients in prognosis”, so it is not really logical to include all these studies into one meta-analysis. For example, in some studies, control group includes healthy people while the other studies used spontaneously recovered HBV patients as control group which makes it very difficult to get one unique conclusion from these studies. However, all these studies were to find and clear one big problem and it is the effect of TNF-*α* gene polymorphism on HBV prognosis (see Table 1).

## 5. Discussion

Past animal researches showed that suppression of HBV protein expression and virus replication are affected by TNF-*α* [53, 54]. Polymorphisms in the promoter of TNF-*α* gene have been accounted to affect the transcription rate and cytokine release. Many studies have attempted to identify the polymorphisms of TNF-*α* promoter and its effect on persistent HBV infection [55, 56]. Nearly 11 single nucleotide polymorphisms (SNPs) in the promoter region of the TNF-*α* gene have been known so far including -163 G/A, -238 G/A, -244 A/G, -308 G/A, -376 G/A, -575 A/G, -857 C/T, -863 C/A, -1031 T/C, -1125 G/C, and -1196 C/T base pairs [51, 57–59]. This polymorphism can affect the TNF-*α* gene transcriptional activity and has different effects on serum TNF-*α* level [60, 61]. While the presence of A allele in -863 locus is associated with a lower serum TNF-*α* level, the presence of C allele in -1031, A allele in -308, and A allele in -238 loci are associated with higher TNF-*α* level in humans [62]. Generally, there is still no worldwide agreement on association between polymorphisms and cytokine production [63]. However, among all these polymorphisms, -308 locus has been studied widely more than the others. 

As mentioned above, there are 3 different attitudes towards the effects of TNF-*α* -308 gene promoter polymorphisms on the prognosis in patients with chronic HBV infection. What can cause these three different results is to some extent clear. The most important factor is the “sample size” issue. Small sample size studies cannot have enough statistical power to show us real accuracy of the expected effects [64–68]. Heterogeneity is in the second level. Different numbers of people from Mongoloid and Caucasoid populations have participated in the studies considering that ethnic differences can play a major role in results from genetic studies [69–71]. As a third reason, different types of HBV patients were observed in the studies including patients with cirrhosis, asymptomatic carriers, and chronic HBV patients. Since we are discussing the genetic effects on prognosis of chronic HBV patients it is important to categorize participations into different stages of HBV infection. Other factors affecting the results including age, lifestyle, environmental factors, family history, and history of other liver diseases in patient should be considered in matching control group with patients. The results from each type of HBV patients should be interpreted and discussed separately especially when observing different conclusions about one unique issue.

These studies may offer the association of TNF-*α* gene polymorphisms with clearance or susceptibility of chronic HBV infection. Noted TNF-*α* gene expression is complex and firmly regulated at the transcriptional, posttranscriptional, and translational levels. However, transcriptional regulation of the TNF-*α* gene has been thought to elucidate molecular mechanisms [72].

## 6. Conclusion

There are many limitations and biases in the discussed studies which make our interpretation difficult to conclude a unique final decision about the effects of TNF-*α* -308 gene promoter polymorphisms on prognosis of chronic HBV infection. We suggest that researchers should design well-defined studies which is better to be a collaborative research program, because of different variations in genetic polymorphism, and it is really important for HBV communities and experts to introduce one or 2 well-designed methods which are able to evaluate the effect of polymorphism on HBV prognosis correctly. Single or separate studies with different methods in this challenging field lead us nowhere.

## Figures and Tables

**Table 1:** Information of TNF-*α* gene polymorphism.

Year	Country	Ethnicity	Chronic HBV Carrier			Recovered HBV infection			Reference
			A/A	G/A	G/G	A/A	G/A	G/G	
2010	Taiwan	Mongoloid	15	38	221	1	39	154	[12]
2010	China	Mongoloid	3	51	250	2	36	323	[13]
2009	South Korea	Mongoloid	1	5	116	1	6	96	[14]
2008	China	Mongoloid	0	6	23	0	15	142	[15]
2008	Turkey	Caucasoid	0	5	45	4	17	39	[16]
2008	China	Mongoloid	3	3	96	5	0	44	[17]
2007	Thailand	Mongoloid	0	22	128	0	18	82	[18]
2007	China	Mongoloid	30	75	40	35	59	6	[19]
2007	Thailand	Mongoloid	0	22	128	1	26	123	[18]
2006	India	Caucasoid	1	65	148	2	136	270	[20]
2006	Iran	Caucasoid	2	20	78	1	20	70	[21]
2006	China	Mongoloid	0	31	91	0	19	44	[22]
2006	South Korea	Mongoloid	1	45	366	1	28	175	[23]
2006	Iran	Caucasoid	2	20	78	1	13	75	[21]
2006	China	Mongoloid	2	15	179	5	10	128	[24]
2005	China	Mongoloid	0	6	125	0	18	108	[25]
2005	Italy	Caucasoid	2	28	154	0	21	75	[26]
2005	China	Mongoloid	0	20	107	0	10	80	[27]
2005	China	Mongoloid	38	72	46	36	48	4	[28]
2005	China	Mongoloid	0	19	203	0	20	83	[29]
2005	China	Mongoloid	2	16	182	5	10	129	[30]
2004	China	Mongoloid	0	6	125	0	22	143	[31]
2003	South Korea	Mongoloid	1	68	971	0	32	251	[32]
2002	Japan	Mongoloid	0	6	207	0	2	50	[33]
2002	China	Mongoloid	0	21	85	0	11	97	[34]
